# Short-term study of atrial shunt and improvement of functional mitral regurgitation

**DOI:** 10.1186/s13019-023-02398-9

**Published:** 2023-11-15

**Authors:** Xiaoke Shang, Mei Liu, Yucheng Zhong, Xueli Wang, Song Chen, Xiaojuan Fu, Ming Sun, Geng Li, Mingxing Xie, Guangyuan Song, Da Zhu, Changdong Zhang, Nianguo Dong

**Affiliations:** 1 Department of Cardiovascular Surgery, Union Hospital, Tongji Medical College, Huazhong University of Science and Technology, 1277 Jiefang Avenue, Jianghan District, Wuhan, 430022 Hubei Province China; 2 Cardiac Laboratory, Department of Cardiovascular Surgery, Union Hospital, Tongji Medical College, Huazhong University of Science and Technology, Wuhan, China; 3 Department of Ultrasound Imaging, Union Hospital, Tongji Medical College, Huazhong University of Science and Technology, Wuhan, China; 4 Heart Valve Disease Intervention Center, Beijing Anzhen Hospital Affiliated to Capital Medical University, Beijing, China; 5 Structural Heart Disease Center, Fuwai Yunnan Cardiovascular Hospital, Kunming, China; 6 Hospital Infection Office, Wuhan No.1 Hospital, Wuhan, China

**Keywords:** Heart failure, Pulmonary hypertension, Interatrial shunt device, Ejection fraction, Trans echocardiography

## Abstract

**Background:**

This study used an atrial septal shunt to compare the treatment progress and prognosis for patients with heart failure (HF) who have different ejection fractions.

**Methods:**

Twenty HF patients with pulmonary hypertension, who required atrial septal shunt therapy, were included in this study. The patients underwent surgery between December 2012 and December 2020. They were divided into two groups based on their ejection fraction: a group with reduced ejection fraction (HFrEF) and a group with preserved ejection fraction(HFpEF) + mid-range ejection fraction (HfmrEF). Echocardiography was utilized to evaluate parameters such as left ventricular dimension (LVD), left ventricular ejection fraction (LVEF), and left ventricular end-diastolic volume (LVEDV). Hemodynamic parameters were measured using cardiac catheterization. The patient's cardiac function was assessed using the six-minute walking test (6MWT), KCCQ score, NYHA classification, and the degree of functional mitral regurgitation (FMR). Followed-up visits were conducted at 1, 3, and 6 months, and any adverse effects were recorded.

**Results:**

The LVEF values were consistently higher in the HFpEF+HFmrEF group than HFrEF group at all periods (*P *< 0.05). Differences in LVD were observed between the two groups before the surgery. Statistically, significant differences were found at the preoperative stage, 1 month, and 3 months (*P *< 0.05, respectively). However, the LVEDV showed a significant difference between the two groups only at 3 months (*P *= 0.049). Notably, there were notable variations in LAPm, LAPs, and the pressure gradient between the LA-RA gradient at baeline, after implantation, and during the 6 months follow-up (all *P *< 0.05).

**Conclusion:**

Following treatment, the HFpEF+HFmrEF group exhibited more significant improvements in echocardiographic and cardiac catheterization indices than the HFrEF group. However, there was no statistically significant difference between the two groups regarding the 6MWT and KCCQ scores. It is important to note that the findings of this study still require further investigation in a large sample size of patients.

## Introduction

Heart failure (HF) is a syndrome characterized by compromised cardiac circulation. It occurs when the heart cannot effectively pump venous blood back to the heart, primarily due to impaired systolic and/or diastolic function. This leads to blood stagnation in the venous system and inadequate blood perfusion in the arterial system [1, 2]. This condition primarily manifests as pulmonary stasis and vena cava stasis. Pulmonary hypertension (PH) is a frequent complication in advanced HF stages and has emerged as a global health concern [3–5].

In order to address the unfavorable prognosis of HF, numerous treatments options are available to manage disease progression and enhance patient survival. Alongside standardized drug therapy, several adjunctive device therapies, such as cardiac resynchronization therapy, implantable monitors, ventricular assist devices, and cardiac transplantation therapies, have emerged to support patient care further [6–8]. An atrial shunt represent a novel approach to treating HF [9, 10]. Elevated left atrial pressure is often the primary factor contributing to clinical symptoms and impaired cardiac function in HF, and it is considered the "ultimate common pathway" leading to various cardiac disease states. By reducing left atrial pressure during rest and exercise, atrial shunts offer a means to alleviate these issues without significantly impacting cardiac output or causing right ventricular failure or pulmonary hypertension. The new shunt act as a pressure regulator, effectively modulating left atrial pressure. Recent clinical studies have reported using an interatrial shunt device (ISD) to treat HF patients [11, 12].

Clinical research has shown that the implantation of an atrial septal shunt device is a safe and effective surgical method for percutaneous treatment of heart failure [13, 14]. This method markedly enhances cardiac output, reduces right heart pressure, and enhances patients’ exercise tolerance, along with other favorable effects on their conditions [15, 16]. Due to its effectiveness in alleviating syncope symptoms in HF, this method has been employed in treating adults suffering from severe and refractory HF, particularly those experiencing recurrent syncopal symptoms [17, 18]. However, more evidence needs to be provided for relevant research due to the limited number of atrial septal shunts utilized in clinical studies. As the technology is still in its early stages of exploration, it is advisable to exercise strict control over patient selection criteria.

Currently, only devices such as cardiac resynchronization therapy (CRTs) and Visia implantable cardioverter defibrillators (ICDs) have been introduced in China, with an annual procedure volume exceeding 4000 cases yearly [19, 20]. However, the high cost of these devices restricts their widespread clinical utilization. In recent years, technology companies such as Medtronic, Abbott, and Boston Scientific have developed some emerging devices for the treatment of heart failure. However, currently, the clinical application of these devices remains very limited. If many HF patients do not receive appropriate treatment, it will adversely impact their survival rate. Consequently, there is a pressing clinical demand for an affordable, safe, and efficient HF treatment. The atrial bypass device has emerged as a novel approach for addressing HF due to its wide-ranging indications, cost-effectiveness, and ease of implementation [21].

In order to contribute novel research insights into HF treatment and gain a comprehensive understanding of the impact of ISD on HF patients with varying ejection fractions, this study employed a controlled clinical trial. The analysis encompassed baseline data, etiology, cardiac catheterization, echocardiography, postoperative cardiac function tests, side effects, and prognosis of HF patients treated with ISD. It is anticipated that these findings will bolster the clinical efficacy of ISD in HF treatment, providing valuable support for its implementation.

## Materials and methods

### Inclusion of patients

All 20 patients diagnosed with left heart failure were obtained from Wuhan Union Hospital. Informed consent was obtained from all patients, who provided their signatures on an informed consent form, and the study received approval from the hospital's ethical committee. The surgical procedures were conducted between December 2012 and December 2020. The patients were divided into two groups according to their ejection fraction values: one group consisted of patients with HF and reduced ejection fraction (HFrEF) (≤ 40%), while the other group comprised patients with HF and preserved ejection fraction (≥ 50%) + mid-range ejection fraction group (40%–50%) (HFpEF + HFmrEF). Each group consisted of 10 patients. The diagnostic criteria for HFrEF [22] encompassed the following parameters: (1) patients aged over 18 years; (2) Individuals with chronic heart failure categorized as New York Heart Association [NYHA] function class II, III, or IV; (3) patients displaying a left ventricular ejection fraction (LVEF) below 40% within 12 months before randomization; (4) patients with elevated levels of the natriuretic peptide within 30 days prior to randomization. For patients in sinus rhythm, the criteria included plasma B-type natriuretic peptide (BNP) levels > 300 pg/mL or NT-proBNP levels > 1000 pg/mL. For patients in atrial fibrillation, the criteria included BNP levels > 500 pg/mL or NT-proBNP levels > 1600 pg/mL. The diagnosis of HFpEF [23] is established according to the American College of Cardiology/American Heart Association (ACC/AHA) consensus guidelines, which comprise the following criteria: (1) presence of typical signs and symptoms of HF in patients with normalcy LVEF within the specified ranges; (2) LVEF ≥ 50%; and (3) absence of other significant predisposing factors for abnormal HF. Exclusion criteria encompass patients with HF who violated the hospital’s medical ethics committee regulations, patients who did not provide informed consent, and individuals with contraindications for surgery. The study has been registered with the Chinese Clinical Trial Registry (No. ChiCTR2000031619).

### Structural composition of the atrial flow regulator and selection of specifications

**Fig. 1:**
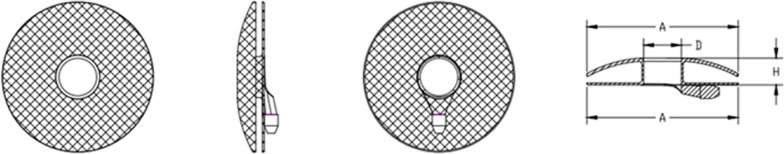
The composition of the atrial septal shunt. All the interatrial shunt devices in this trial came from Wuhan Weike Medical Technology Co., Ltd (Wuhan, China)

The atrial flow regulator comprises a nickel-titanium alloy woven mesh plug, a diverter hole, and an eccentric transverse stainless steel nut end (Fig. 1). Table 1 provides details regarding various sizes of atrial bypass devices. The D-shant atrium shunt device was designed in four sizes according to clinical requirements: 4, 6, 8, and 10 mm, which corresponded to the device's disc diameter: 16, 20, 24, and 28 mm [14]. In this trial, all interatrial shunt devices were categorized into four types: WKASD16-4, WKASD20-6, WKASD20-8, and WKASD24-10 (Wuhan Weike Medical Technology Co., Ltd, Wuhan, China).

**Table 1:** Details of several specifications of atrial bypass devices

Specification code	Diameter of the divided-flow hole at the waist (mm)	The diameter of the intervertebral disc surface (mm)	Device thickness (mm)
WKASD28-10	10	28	6.5
WKASD24-8	8	24	6.5
WKASD20-6	6	20	6.5
WKASD16-4	4	16	6.5

### Ultrasound guidance and monitoring during atrial bypass placement

**Fig. 2:**
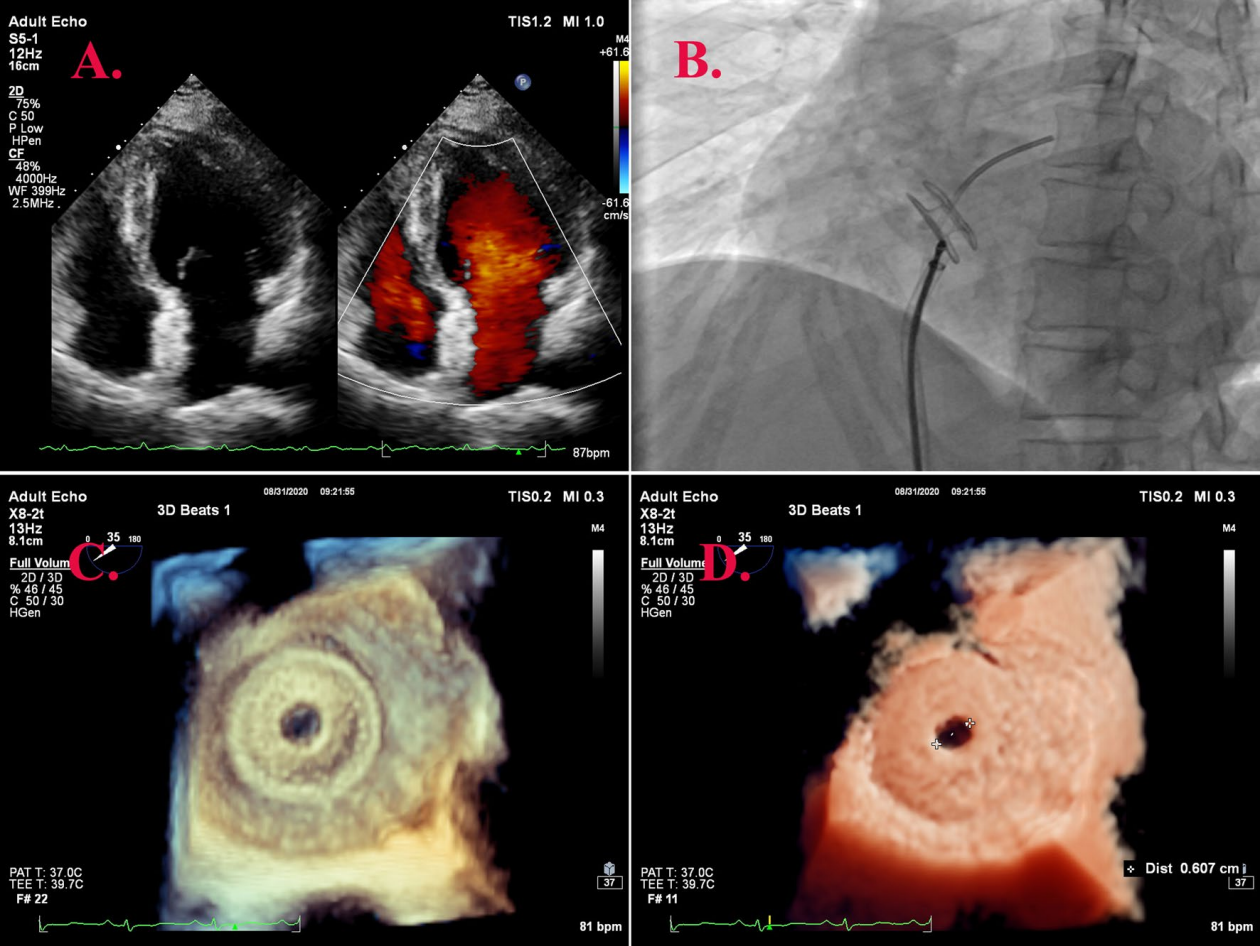
Color Doppler shows the shunt signal. **A** Echocardiogram showing D-shant atrial shunt discs tightly clamped on both sides of the atrial septum. Two-dimensional ultrasound findings are shown on the left and colour Doppler ultrasound findings are on the right. **B** Immediate left atrial pressure changes measured by catheterization after atrial shunt implantation in the atrial septum. **C** 3D transesophageal echocardiography (3D TEE) clear reconstruction of D-shant's left atrial disk slice. **D** The shunt orifice diameter of the D-shant was 0.6 cm measured under 3D TEE

Prior to commencing the procedure, transesophageal ultrasound was conducted from multiple angles. This examination ruled out the presence of intra-cardiac thrombus, atrial septal defect, and atrial septal tumor, confirming the suitability of the indications. Following the puncture, a pre-shaped stiffened wire was guided into the left atrium through the septal puncture site. Subsequently, a 10 mm peripheral arterial balloon was advanced to the septum for repeated balloon dilation at 8 standard atmospheres. Real-time ultrasound monitoring visualized the puncture site, path, and dilation size. The balloon was then retracted and transferred to the delivery system, where a 20–6 mm D-shant atrial shunt was selected. The shunt was released on both sides of the atrial septum under the guidance of transesophageal ultrasound monitoring. The transesophageal ultrasound confirmed a normal shunt morphology and position, with an approximate shunt orifice diameter of 6 mm and a clear left-to-right shunt signal. Three-dimensional ultrasound was employed to observe the shape of the shunt and its relationship with surrounding tissues. The left atrial pressure decreased from 15 mmHg before shunt placement to 8 mmHg after shunt release (Fig. 2). The patient was transferred to the monitoring ward for postoperative observation after the procedure.

### Ultrasound examination

This study used thoracic echocardiography (TTE) and transesophageal echocardiography (TEE) to evaluate the implanted atrial septal shunt. The patients' left ventricular dimension (LVD, cm), LVEF, and left ventricular end-diastolic volume (LVEDV, ml/m^2^) were also measured. The term average loss of aperture is calculated as the difference between the measured orifice diameter by TEE immediately after the surgery and the estimated orifice diameter by TEE at the endpoint, divided by the measured orifice diameter by TEE immediately after the surgery. Procedural success is defined as the patient achieving a shunt orifice diameter essentially in line with the expected product specifications (with a difference of less than 2 mm). Additionally, it involves an immediate reduction of at least 3 mmHg in mLAP/mPCWP (mean left atrial pressure/mean pulmonary capillary wedge pressure) and the patient surviving the perioperative period. The perioperative survival rate is defined as the patient survival rate within 30 days after surgery.

### Cardiac catheterization

Cardiac catheterization was employed to measure various hemodynamic indices in the HFrEF and HFpEF+HFmrEF groups. These included the mean left atrial pressure (LAPm), left atrial systolic pressure (LAPs), mean right atrial pressure (mRAP), mean gradient between the left atrium and right atrium (LA-RA gradient), pulmonary artery systolic pressure (PASP), pulmonary-to-systemic flow ratio (Qp/Qs), and cardiac index (CI).

### Cardiac function tests

This study evaluated cardiac function in both groups using various assessment tools, including the 6-min walking test (6MWT) [24], Kansas City Cardiomyopathy Questionnaire (KCCQ) score [25], New York Heart Association (NYHA) classification, and functional mitral regurgitation (FMR) [26]. The guidelines for conducting the 6MWT have been published and regularly updated by the American Thoracic Society (ATS). This test applies to patients with moderate or severe cardiac insufficiency, older or frailer patients, obese patients, and patients with permanent pacemakers. The KCCQ is a self-administered questionnaire comprising 23 items (15 questions) to quantify physical limitations, symptoms (frequency, severity, and recent changes over time), social limitations, self-efficacy, and quality of life. All items are measured using a Likert scale, which includes 5–7 response options. The questionnaire comprises five individual subscales. Except for the self-efficacy subscale, all subscales are aggregated to generate clinical and overall summary scores. The scores for each subscale are standardized on a scale from 0 to 100, with higher scores indicating better health status, fewer symptoms, and higher disease-specific Health-Related Quality of Life (HRQoL) [27, 28]. The NYHA classification categorizes the degree of impaired cardiac function into four classes based on the activity level at which HF symptoms are induced. The classification of FMR is divided into five classes. The grading criteria for FMR are referenced from the American Society of Echocardiography guidelines and are divided into five grades (0 to 4) [29].

### Statistical analysis

The data in this study were collected and analyzed using SPSS 25.0 software. The t-test was employed for comparing two groups of continuous data that followed a positive-terrestrial distribution. At the same time, the paired t-test was used for paired data groups that also adhered to paired information. Analysis of variance was utilized for comparing multiple groups of data that followed an orthogonal distribution. Non-parametric tests, such as the Kruskal–Wallis H test or Mann–Whitney U test, were applied for continuous data that did not exhibit a positive-terrestrial distribution. The chi-square test was utilized for analyzing count data. A significance level of *P *< 0.05 was considered statistically significant.

## Results

### Baseline information

A total of 20 patients with left heart failure requiring atrial bypass treatment were included in this study. They were divided into two groups based on their ejection fraction: the HFrEF group (10 patients) and the HFpEF+HFmrEF group (10 patients). In the HFrEF group, six patients (60%) were male, with an average age of 57.0 ± 11.1 years. Similarly, in the HFpEF+HFmrEF group, six patients (60%) were male, averaging 61.6 ± 11.8 years. There was no statistically significant difference in gender and age distribution between the two groups (*P *> 0.05). However, there was a significant difference in BMI between the groups (*P *= 0.046), while no significant difference was observed in body surface area (BSA) (*P *= 0.110) (Table 2).

**Table 2:** Baseline data results for both groups

Groups	HFpEF + HFmrEF (n = 10)	HFrEF (n = 10)	*P*
Gender (male/female)	6/4	6/4	1.000
Age (years)	57.0 ± 11.1	61.6 ± 11.8	0.380
BMI (kg/m^2^)	25.1 ± 3.6	21.9 ± 3.7	0.046
BSA (m^2^)	1.89 ± 0.26	1.71 ± 0.19	0.110
Etiology			
DHF	1	0	
DCM	1	0	
HFFMI	5	4	
HC	2	0	
FDC	1	0	
DCM	0	6	
Smoking	4/10	3/10	1.000
Alcohol	4/10	4/10	1.000
Hypertension	3/10	0/10	0.211
Diabetes	4/10	5/10	1.000
Hyperlipidemia	3/10	3/10	1.000
Atrial fibrillation/flutter	4/10	1/10	0.303
CHD	5/10	5/10	1.000
Stroke	0/10	0/10	1.000
PVD	1/10	0/10	1.000
RI	0/10	1/10	1.000

*DHF* Diastolic heart failure, *DCM*: Diabetic cardiomyopathy, *HC*: Hypertensive cardiomyopathy, *HFFMI*: Heart failure following myocardial infarction, *FDC*: familial dilated cardiomyopathy, *DCM*: dilated cardiomyopathy, *CHD*: coronary heart disease, *PVD*: Peripheral Vascular Disease, *RI*: renal insufficiency

Furthermore, there were no statistically significant differences between the two groups in terms of smoking, alcohol consumption, and underlying diseases such as hypertension, diabetes, hyperlipidemia, atrial fibrillation/flutter, coronary heart disease (CHD), stroke, peripheral vascular disease (PVD), and renal insufficiency (RI) (all *P* values > 0.05). Detailed results can be found in Table 2.

Among the patients included in this study, the etiological studies of pulmonary hypertension revealed the following distribution: one case of diastolic heart failure (DHF), one case of dilated cardiomyopathy (DCM), five cases of heart failure following myocardial infarction in the HFpEF+HFmrEF group (HFFMI), two cases of hypertensive cardiomyopathy (HC), and one case of familial dilated cardiomyopathy (FDC) in the HFpEF+HFmrEF group. In the HFrEF group, there were four cases of heart failure following myocardial infarction (HFFMI), along with six cases of dilated cardiomyopathy (DCM). Detailed results can be found in Table 2.

Regarding the surgical results, we assessed the implanted atrial septal shunt postoperatively using both thoracic echocardiography (TTE) and transesophageal echocardiography (TEE). The results demonstrated a minimal average reduction in aperture size, with only 1% for TEE and 4% for TTE. The procedure was successful in 100% of cases, with a perioperative patient survival rate of 100% and a significant reduction in left atrial pressure of 7.85 ± 2.72 mmHg.

### Hemodynamics

**Fig. 3:**
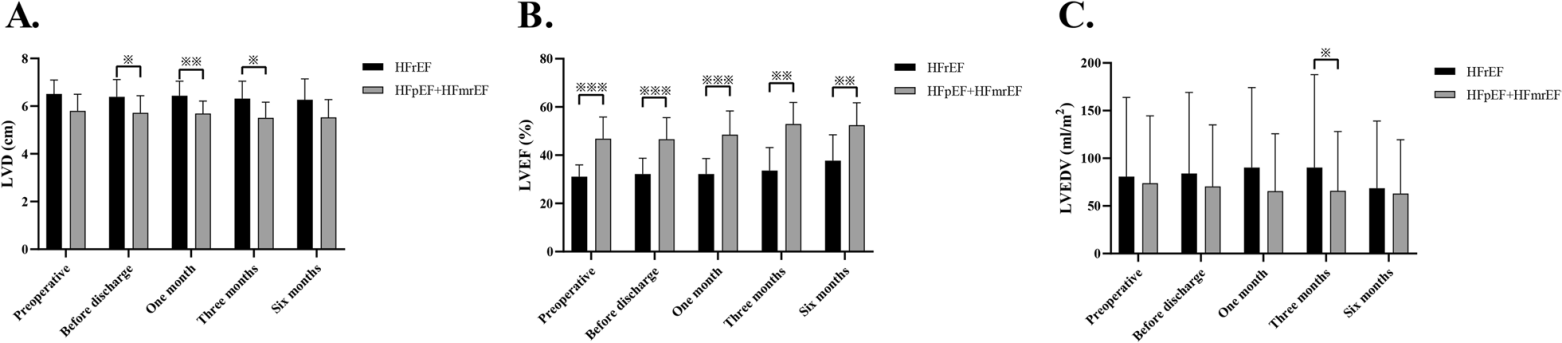
LVD (cm), LVEF and LVEDV (ml/m^2^) values at different time points in the HFrEF, and HFpEF+HFmrEF groups. **A** LVD (cm); **B** LVEF; **C** LVEDV (ml/m^2^). ※: *P *< 0.05, ※※: *P *< 0.01, ※※※: *P *< 0.001

The pre-and post-operative hemodynamics assessed by cardiac catheterization showed no statistically significant difference in left ventricular diastolic diameter (LVD) between the periods in the HFrEF group (*P *= 0.950). Similarly, there was no statistical difference in LVD between the periods in the HFpEF+HFmrEF group (*P *= 0.843). When comparing LVD values between the HFrEF and HFpEF+HFmrEF groups for the same period, statistical differences were observed at the preoperative, 1-month, and 3-month time points (*P *= 0.024, 0.009, and 0.026, respectively). However, there was no statistically significant difference in LVD values between the two groups at the time of preoperative and six months (*P *= 0.052, 0.063, respectively) (Fig. 3A).

 HFpEF+HFmrEF In the different time periods, there was no statistically significant difference in LVEF values within both the HFrEF and HFpEF+HFmrEF groups (*P *= 0.394 and 0.372, respectively). However, LVEF values in the HFpEF+HFmrEF group were consistently higher than those in the HFrEF group during the same period, and these differences were statistically significant (*P *< 0.001, 0.001, < 0.001, 0.005, and 0.005, respectively) (Fig. 3B).

The comparison of LVEDV values between the HFrEF and HFpEF+HFmrEF groups for the same period showed a statistical difference only at three months (*P *= 0.049), while no statistical differences were observed for the remaining periods (*P *= 0.421, 0.218, 0.171, and 0.382, respectively) (Fig. 3C).

### The results of cardiac catheterization

Hemodynamic parameters, including mean left atrial pressure (LAPm), left systolic atrial pressure (LAPs), mean right atrial pressure (mRAP), LA-RA gradient, pulmonary artery systolic pressure (PASP), Qp/Qs, and cardiac index (CI), were measured using cardiac catheters in both the HFrEF and HFpEF+HFmrEF groups. Statistical analysis revealed significant differences in LAPm, LAPs, mRAP, and LA-RA gradient between baseline (n = 10), post-implantation (n = 10), and the 6-month follow-up (n = 7) in the HFrEF group (all *P *< 0.05), and three patients did not undergo cardiac catheterization at 6 months. In the HFpEF+HFmrEF group, statistical differences were found in LAPm, LAPs, and LA-RA gradients at different time points (all *P *< 0.05), and two patients did not undergo cardiac catheterization at 6 months. However, no statistical differences were observed among the remaining indicators (*P *> 0.05). For detailed results, please refer to Table 3.

**Table 3:** The results of cardiac catheterization

Indicator	HFrEF			P	HFpEF+HFmrEF			*P*
	Baseline (n = 10)	After implantation (n = 10)	6 months follow-up (n = 7)		Baseline (n = 10)	After implantation (n = 10)	6 months follow-up (n = 8)	
LAPm	18.6 ± 4.7	11.6 ± 3.4	8.1 ± 5.4	** < 0.001**	17.0 ± 2.4	8.8 ± 3.2	9.3 ± 5.8	** < 0.001**
LAPs	29.7 ± 12.4	18.8 ± 7.4	13.6 ± 5.4	**0.004**	24.7 ± 8.0	15.1 ± 6.1	18.3 ± 10.3	**0.042**
mRAP	6.3 ± 1.6	5.5 ± 2.3	2.9 ± 2.3	**0.008**	4.8 ± 1.5	4.4 ± 1.8	4.5 ± 4.0	0.937
LA-RA gradiant	12.3 ± 3.9	6.1 ± 3.5	5.3 ± 3.8	**0.001**	12.2 ± 1.8	4.4 ± 2.1	4.8 ± 2.1	** < 0.001**
PASP-	44.3 ± 20.5	38.8 ± 16.4	16.6 ± 5.6	0.174	42.9 ± 10.6	36.9 ± 10.6	32.1 ± 12.2	0.139
Qp/Qs	1.0	1.35 ± 0.14	1.35 ± 0.14	0.949	1.0	1.33 ± 0.13	1.35 ± 0.16	0.804
CI	2.0 ± 0.7	2.3 ± 0.8	2.2 ± 0.4	0.591	2.0 ± 0.5	2.1 ± 0.5	2.4 ± 0.7	0.467

Bold value indicates *P *< 0.05

### Cardiac function tests

**Fig. 4:**
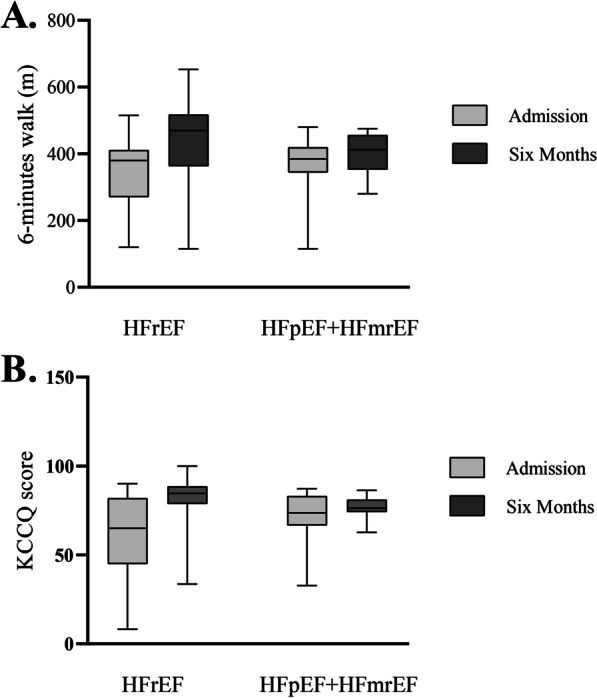
Testing of cardiac function. **A** 6-min walking test; **B** KCCQ score

The median six-minute walk distance was 380 (270, 412) in the HFrEF group and 385 (344, 420) in the HFpEF+HFmrEF group, with no statistically significant difference observed between the two groups (*P *= 0.762) (Fig. 4A). The median value of the KCCQ score test was 65.0 (44.8, 82.1) in the HFrEF group and 73.6 (66.6, 83.2) in the HFpEF+HFmrEF group, with no statistically significant difference found between the two groups (*P *= 0.405) (Fig. 4B).

**Fig. 5:**
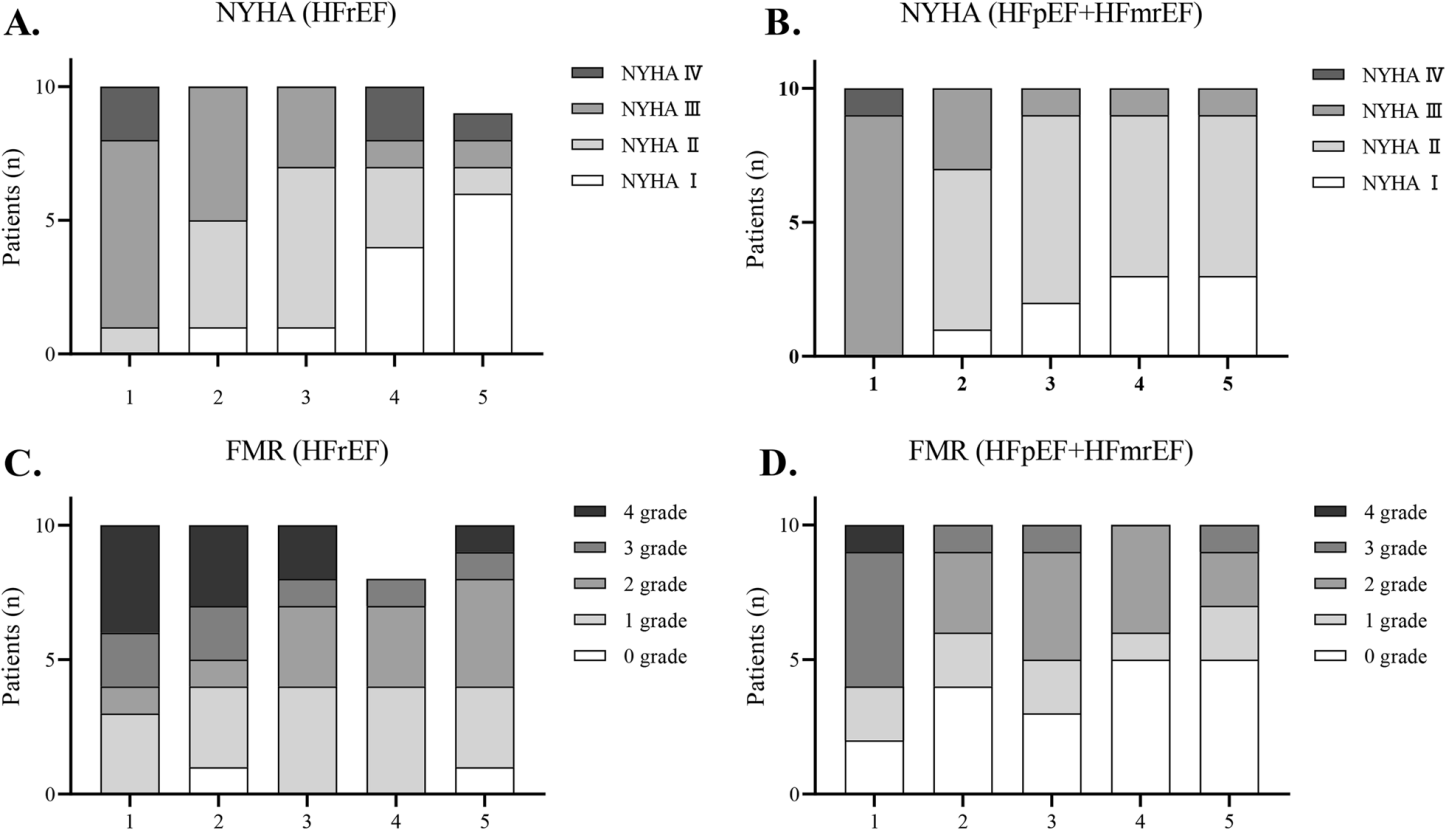
The NYHA classification and FMR classification of patients at different times. **A** NYHA classification for HFrEF group; **B** NYHA classification for HFpEF group; **C** FMR classification for HFrEF group; **D** FMR classification for HFpEF+HFmrEF group

Regarding the NYHA classification, in the HFrEF group, one patient had grade II before admission, seven had grade III, and two had grade IV. After six months of follow-up, there were six patients with grade I, one with grade II, one with grade III, and one with grade IV (Fig. 5A). In the HFpEF+HFmrEF group, nine patients had grade III and one with grade IV before admission. After six months of follow-up, three patients had grade I, six had grade II, and one had grade III (Fig. 5B).

For functional mitral regurgitation (FMR), in the HFrEF group, there were three patients with grade 1, one patient with grade 2, two patients with grade 3, and four patients with grade 4 before treatment; there was one patient with grade 0, three with grade 1, four with grade 2, one with grade 3, and one with grade 4 after six months (Fig. 5C). In the HFpEF+HFmrEF group, there were two patients with grade 0, two with grade 1, five with grade 3, and one with grade 4. After six months of follow-up, there were five patients with grade 0, two with grade 1, two with grade 2, and one with grade 3 in the FMR (Fig. 5D).

### Adverse effects and follow-up

All patients underwent the procedure under local anesthesia, and no procedure-related complications were observed. Additionally, there were no orifice stenosis occlusion, embolic events, or other adverse events. A 6-month follow-up was completed by all but one patient who was lost to follow-up. Five patients experienced postoperative cardiovascular adverse events, including new-onset atrial flutter atrial fibrillation, temporary postoperative hypotension in patients with class IV cardiac function, recurrent heart failure, and rehospitalization with abnormal ICD discharge in fast ventricular rate atrial fibrillation.

## Discussion

According to the different ejection fractions, the patients included in this study were divided into the HFrEF and HFpEF+HFmrEF groups. The statistical analysis showed no significant differences in LVD, LVEF, and LVEDV within each group at different times (all *P *> 0.05). The detection of LVD at the same time points between the groups showed that the values in the HFrEF group were higher than those in the HFpEF group before surgery, at one month and three months (all *P *< 0.05). A comparison of LVEF values showed that the HFpEF+HFmrEF group consistently had higher values than the HFrEF group (all *P *< 0.05). A comparison of LVEDV values showed that only in the third month was the LVEDV value in the HFpEF+HFmrEF group higher than that in the HFrEF group (*P *< 0.05). Statistical analysis of hemodynamic parameters showed significant differences in LAPm, LAPs, mRAP, and LA-RA gradient between baseline, post-implantation, and 6-month follow-up in the HFrEF group (all *P *< 0.05). The HFpEF group showed statistically significant differences in LAPm, LAPs, and LA-RA gradient at different time points (all *P *< 0.05). Although there was no statistical difference in the six-minute walking distance test between the two groups (*P *> 0.05), both groups showed improvement in NYHA classification and FMR detection after treatment.

Pulmonary hypertension (PH) is a prevalent complication of end-stage heart failure [30]. The conventional treatment for this condition relies on targeted drugs, interventions, and surgery [31]. However, targeted drug therapy presently only alleviates symptoms in a subset of patients, and this medications are costly and fall short of providing optimal treatment for PH [32].

In addition to pharmacologic therapy, clinical treatments for pulmonary hypertension of HF encompass various procedures such as balloon angioplasty, balloon atrial septostomy, percutaneous stent implantation, atrial septostomy, and lung transplantation [33]. Balloon angioplasty, a widely recognized technique, aims to create or expand atrial communication to enhance cardiac output in diverse scenarios. However, this approach is accompanied by potential complications, including early fenestration closure, oversaturation, stent occlusion or migration, and challenges in adjusting the shunt size to achieve the desired hemodynamic effect [34]. Atrial septostomy and atrial septal stent implantation are recognized as established techniques to prevent early fenestration closure, although the surgical procedure involved is more intricate [35]. The complexity of the procedure is amplified by the necessity for repeated catheter and balloon exchanges during balloon atrial septostomy (BAS), which escalates the surgical risk. Additionally, patients undergoing these interventions face a considerable surgical mortality rate, with early postoperative spontaneous closure of the opening frequently occurring. While the procedure can prevent spontaneous closure, there exists a significant risk of hypoxia. Stent embolization cannot be averted, leading to uncontrollable stoma diameter and irregularity, which can easily result in early occlusion. Consequently, BAS serves solely as a late palliative treatment in HF patients [36]. Furthermore, lung or combined heart–lung transplantation is exclusively employed for managing advanced pulmonary hypertension [37]. However, in recent years, atrial septal shunts with a right-to-left shunt have garnered clinical acceptance as a treatment option for pulmonary hypertension.

In recent years, the interatrial shunt device (ISD) has emerged as a promising technology for treating HF internationally. Several types of atrial shunts are currently employed in clinical practice, including Corvia IASD [38], V-Wave Shunt [39], and Occlutech AFR [40]. Compared to traditional device treatment modalities, this approach offers numerous advantages, such as broad indications, economical safety, and easy implementation [21]. While targeted drug therapy does enhance survival, exercise capacity, and quality of life (QoL) for HF patients, it is an expensive option that may not be suitable for all individuals with HF. In some cases, this treatment method may even accelerate disease progression [41]. Conversely, establishing an atrial septal right-to-left shunt has proven highly effective in alleviating symptoms of right HF, particularly in idiopathic HF [42]. By controlling the diameter of the atrial septal stoma, the device relieves pulmonary stasis and dyspnea while effectively reducing left atrial pressure. Importantly, it does not significantly increase the right heart burden, reduce cardiac output, or contribute to the development of paradoxical embolism. ISD represents a novel alternative for managing clinical symptoms of HF, including decreased exercise capacity, syncope, or significant right heart failure. It can be utilized in adults with severe and resistant pulmonary hypertension, especially in patients experiencing recurrent syncopal symptoms [40, 43].

The clinical utilization of ISD was initially pioneered by Micheetti et al. [44] in 2006. Their approach involved implanting a custom-made open septal device at the septal stoma end, effectively preventing early closure of the ASD triggered by static balloon dilation, thus maintaining stoma patency. In their study, which included 20 pediatric patients, 7 children exhibited a short-term stoma non-closure incidence of 35%. Similarly, Lammers et al. [45] reported on 10 cases, 7 involving severe PAH, where an Amplatzer atrial septal defect sealer with a window was implanted. Postoperatively, patients received warfarin, aspirin, or heparin to prevent thrombus formation at the open window site. Ultimately, all PAH patients in this study experienced symptom relief. However, during the subsequent follow-up period of up to 10 months, a notable occurrence of window occlusion was observed in 4 patients, resulting in an occlusion rate of 40%. This modified Amplatzer septal sealer, featuring a custom window opening, demonstrated a high re-occlusion rate. The underlying reason for this is that the windowed septal occluder merely creates a hole in the central portion of the waist, with the edges of this hole serving as a common site for thrombus aggregation. Despite administering anticoagulants like warfarin, the occlusion rate remains high due to the hypercoagulable state of blood in patients with PH.

A total of 20 patients with HF requiring treatment with an interatrial shunt device were enrolled in this study. Based on their ejection fraction, the patients were divided into two groups: a reduced ejection fraction group of 10 patients and a preserved ejection fraction + mid-range ejection fraction group comprising another 10 patients. This division allowed for the observation and investigation of the prognosis of patients with different ejection fractions undergoing treatment with an interatrial shunt device for arterial hypertension (AH). Regarding the comparison of baseline data, only the difference in BMI (body mass index) showed statistical significance between the two groups (*P *= 0.46). Other factors such as age, gender, and BSA (body surface area) did not exhibit statistically significant differences between the two groups (all *P* values > 0.05). The increased BMI in the preserved + mid-range ejection fraction group is most likely associated with the extensively studied "obesity paradox" phenomenon in cardiovascular disease [46, 47]. Naturally, it is essential to note that this conclusion may be influenced by the limited number of patients included in the study. Nevertheless, no statistically significant differences were observed between the two groups regarding adverse lifestyle habits such as smoking and alcohol consumption and underlying conditions, including hypertension, diabetes, and hyperlipidemia. These findings indicate a high level of comparability between the two patient groups in the study.

Echocardiography was employed to measure the left ventricular diameter (LVD), left ventricular ejection fraction (LVEF), and left ventricular end-diastolic volume (LVEDV) in both patient groups at five different time points (preoperative, before discharge, one month, three months, and six months) (all *P *> 0.05). However, statistical differences were observed between the two groups during specific periods. Specifically, LVEF values were consistently higher in the HFpEF+HFmrEF group than in the HFrEF group across all time points (all *P *< 0.05). Additionally, statistically significant differences in LVD indicators were observed between the two groups at the preoperative, 1-month, and 3-month follow-ups (*P *= 0.024, 0.009, and 0.026, respectively). Conversely, there was a statistical difference in the LVEDV indicator only at the 3-month follow-up (*P *= 0.049).

These findings underscore the effectiveness of LVEF as a dependable predictor for distinguishing between the two patient groups, both during surgery and in the pre-treatment period. Moreover, LVD is a valid indicator for comparing the two groups in the pre-treatment phase. However, in the current study, LVEDV exhibited limited utility in distinguishing between the two groups. This is the same result as the study by Ahmed Hussein Subki et al. [48]. The study demonstrated that patients with HFrEF exhibited higher left ventricular diastolic volume (LVD) (1536 vs. 826), left ventricular systolic volume (LVs) (1660 vs. 772), and left atrial volume (1344 vs. 875) compared to patients with HFpEF+HFmrEF (*P *< 0.05).

We evaluated cardiac function in both groups using the 6-min walk test (6MWT), Kansas City Cardiomyopathy Questionnaire (KCCQ) score, New York Heart Association (NYHA) classification, and functional mitral regurgitation (FMR). However, no statistically significant differences between the two groups regarding 6MWT and KCCQ scores (all *P *> 0.05) were observed. In the NYHA classification, prior to admission, the HFrEF group consisted of seven patients in grade III and one in grade IV. After six months of follow-up, one patient remained in grade III, and two remained in grade IV. Regarding functional mitral regurgitation (FMR), the HFrEF group initially had two patients in grade 3 and four in grade 4. After six months of follow-up, one patient was in grade 3, and one was in grade 4. For the HFpEF+HFmrEF group, nine patients were in grade III and one in grade IV prior to admission, according to the NYHA classification. After six months of follow-up, only one patient remained in grade III. Regarding FMR, the HFpEF group initially had five patients in grade 3 and one in grade 4. After six months of follow-up, only one patient remained in grade 3. These findings suggest that patients in the HFpEF+HFmrEF group had better prognoses than those in the HFrEF group, as indicated by the NYHA and FMR classifications. However, given the limited number of patients included in the study, further support from additional clinical data is required to strengthen this conclusion.

Although this study effectively treated heart failure patients using atrial septal defect closure, some limitations remain. For instance, the need for more patients will limit the reliability of the research results. Additionally, we did not evaluate right heart catheterization and pulmonary status before the procedure. Furthermore, including heart failure patients with different etiologies will increase the heterogeneity of the study.

## Conclusion

The limited number of patients included in this study imposes certain limitations on the conclusions that can be drawn. Cardiac ultrasonography revealed that the HFpEF+HFmrEF group demonstrated better LVEF (%), LVD indexes, LAPm, LAPs, and LA-RA gradient than the HFrEF group following atrial shunt treatment. However, the two groups had no significant differences regarding the 6MWT and KCCQ. Based on the study mentioned above and analysis, it can be concluded that atrial septal shunt treatment showed superiority in HFrEF compared to HFpEF+HFmrEF, particularly in cardiac ultrasound assessment. However, HFpEF+HFmrEF did not demonstrate significant superiority over HFrEF in improving cardiac function. The findings of this study warrant further investigation in a larger sample size of patients.

## Data Availability

The datasets generated and analyzed during the current study are available from the corresponding author on reasonable request.
